# Achievement motivation modulates Pavlovian aversive conditioning to goal-relevant stimuli

**DOI:** 10.1038/s41539-019-0043-3

**Published:** 2019-04-24

**Authors:** Yoann Stussi, Aude Ferrero, Gilles Pourtois, David Sander

**Affiliations:** 1 Swiss Centre for Affective Sciences, Campus Biotech, University of Geneva, Geneva, Switzerland; 2 Laboratory for the study of Emotion Elicitation and Expression (E3Lab), Department of Psychology, University of Geneva, Geneva, Switzerland; 3 Cognitive & Affective Psychophysiology Laboratory (CAP-lab), Department of Experimental Clinical & Health Psychology, Ghent University, Ghent, Belgium

**Keywords:** Classical conditioning, Human behaviour, Emotion

## Abstract

Pavlovian aversive conditioning is a fundamental form of learning helping organisms survive in their environment. Previous research has suggested that organisms are prepared to preferentially learn to fear stimuli that have posed threats to survival across evolution. Here, we examined whether enhanced Pavlovian aversive conditioning can occur to stimuli that are relevant to the organism’s concerns beyond biological and evolutionary considerations, and whether such preferential learning is modulated by inter-individual differences in affect and motivation. Seventy-two human participants performed a spatial cueing task where the goal-relevance of initially neutral stimuli was experimentally manipulated. They subsequently underwent a differential Pavlovian aversive conditioning paradigm, in which the goal-relevant and goal-irrelevant stimuli served as conditioned stimuli. Skin conductance response was recorded as an index of the conditioned response and participants’ achievement motivation was measured to examine its impact thereon. Results show that achievement motivation modulated Pavlovian aversive learning to goal-relevant vs. goal-irrelevant stimuli. Participants with high achievement motivation more readily acquired a conditioned response to goal-relevant compared with goal-irrelevant stimuli than did participants with lower achievement motivation. However, no difference was found between goal-relevant and goal-irrelevant stimuli during extinction. These findings suggest that stimuli that are detected as relevant to the organism can induce facilitated Pavlovian aversive conditioning even though they hold no inherent threat value and no biological evolutionary significance, and that the occurrence of such learning bias is critically dependent on inter-individual differences in the organism’s concerns, such as achievement motivation.

## Introduction

Pavlovian aversive conditioning is a fundamental form of learning in the animal kingdom, being ubiquitous across a wide variety of species ranging from simple (e.g. fruit fly) to more complex (e.g. human) organisms.^1^ It consists of both the learning process and procedure whereby an environmental stimulus (the conditioned stimulus) acquires the ability to elicit a preparatory response (the conditioned response) by virtue of a single or repeated contingent pairing with a biologically significant aversive event (the unconditioned stimulus).^2,3^ However, not all stimuli are equally associable in Pavlovian aversive conditioning.^4^ Previous research has shown that specific classes of evolutionarily threat-relevant stimuli, such as snakes, angry faces, or outgroup faces, are more rapidly^5,6^ and persistently^6–9^ associated with an aversive outcome than nonthreatening stimuli, such as flowers, happy faces, or ingroup faces.^10,11^ These preferential associations have generally been interpreted as evidence for the preparedness^12^ and fear module^11^ theories, which posit that organisms are biologically prepared to associate stimuli that have posed threats to the species’ survival across evolution with aversive events.

At variance with these evolutionary theories, an alternative framework deriving from appraisal theories of emotion^13,14^ asserts that preferential emotional learning is not driven by a threat-specific mechanism, but by a more general mechanism of relevance detection.^15,16^ Relevance detection is a rapid and adaptive process that determines whether a stimulus encountered in the environment is relevant to the organism’s concerns, such as their goals, needs, motives, or values.^13,14,16–18^ Importantly, this proposal allows for incorporating the findings of preferential Pavlovian aversive conditioning to threat-relevant stimuli, as these stimuli are highly relevant for the organism’s survival, but also generates new testable predictions: Stimuli that are detected as relevant to the organism’s concerns benefit from preferential emotional learning, regardless of their valence and evolutionary status per se.

In agreement with this hypothesis, we have recently shown that, similar to threat-relevant stimuli (angry faces and snakes), positive stimuli with biological relevance (baby faces and erotic stimuli) are likewise persistently associated with an aversive outcome (electric stimulation) during Pavlovian aversive conditioning, thereby demonstrating that preferential Pavlovian aversive conditioning is not restricted to negative threat-related stimuli but extends to positive relevant stimuli.^16^ Nonetheless, this study did not address the question of whether the stimulus’ evolutionary history might be a key ingredient in this preferential emotional learning. Existing evidence on this issue is mixed: whereas some studies found a similar enhanced resistance to extinction of learned threat to both biological (snakes) and cultural (pointed guns) threats,^19,20^ other studies observed a greater persistence of learned threat to threat-relevant stimuli from phylogenetic origin than from ontogenetic origin.^21,22^ Accordingly, whether enhanced emotional learning is confined to evolutionarily relevant stimuli or encompasses stimuli with high relevance to the organism beyond biological and evolutionary considerations remains to be better elucidated.

A key assumption of the relevance detection model is that emotional learning is largely affected by individual differences in affect and motivation. The process of relevance detection is inextricably tied to the organism’s concerns, the salience and priority of which may flexibly and rapidly change based on current environmental contingencies, and which are likely to vary across individuals.^14,17,23^ As a result, the same stimulus may potentially produce a learning bias for a given individual, but not for another one, if these two individuals differ according to their current concerns, and hence the way in which they appraise the stimulus at stake. In line with this view, inter-individual differences are inherent and highly prevalent in Pavlovian conditioning,^2,24^ as reflected by a substantial variability across individuals in this learning process as a function of biological, experiential, or personality factors, as well as affective or cognitive biases.^24–30^ Despite these initial attempts to consider inter-individual differences for yielding a better understanding of emotional learning in humans, their contribution to Pavlovian conditioning, along with the underlying mechanisms thereof, remain yet poorly understood.^24^

**Fig. 1:**
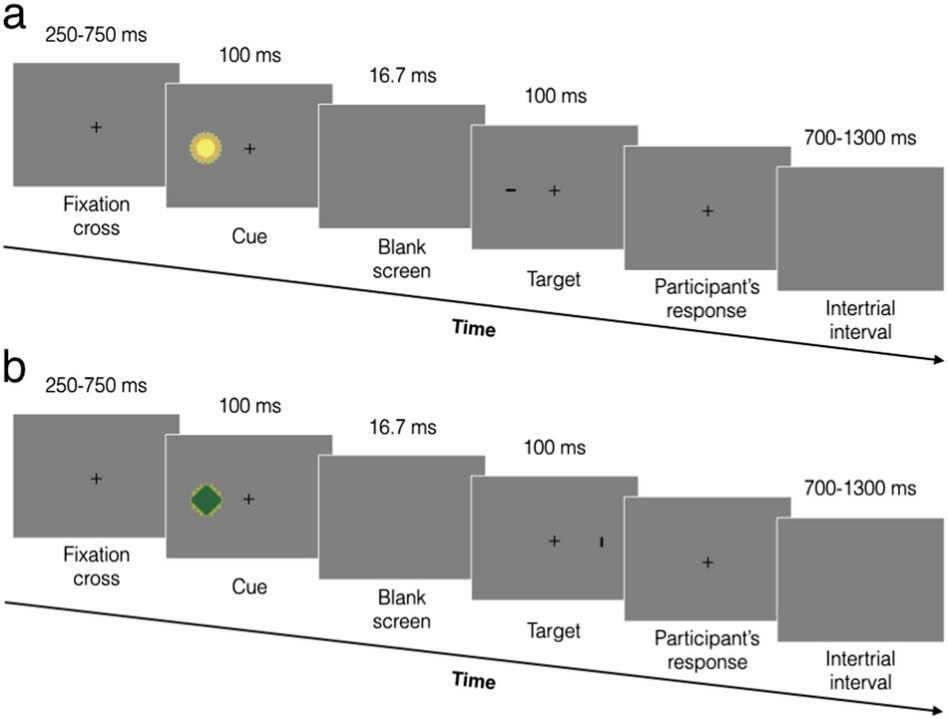
Illustration of the spatial cueing task used in the experiment. **a** In valid trials, the target appeared at the same location as the cue. **b** In invalid trials, the target appeared at the opposite location as the cue. The cues were geometric figures, which systematically predicted target location at the same (goal-relevant valid) or the opposite (goal-relevant invalid) location, or were nonpredictive of target location (goal-irrelevant). Participants were requested to detect the target orientation (horizontal vs. vertical)

Here, we therefore aimed to investigate whether enhanced emotional learning could occur to stimuli that are relevant to the organism’s concerns independently of their intrinsic evolutionary significance, as well as the modulatory role of inter-individual differences therein. To this end, we used initially neutral stimuli (i.e. geometric figures) and experimentally manipulated their relevance for task goals in a spatial cueing task^31^ (Fig. 1), some stimuli being goal-relevant by predicting target location (goal-relevant valid stimuli) or predicting the opposite location relative to the target (goal-relevant invalid stimuli), and others goal-irrelevant by being nonpredictive of target location (goal-irrelevant stimuli). We subsequently used these stimuli as conditioned stimuli (CS) in a differential Pavlovian aversive conditioning paradigm. In this paradigm, one stimulus (CS+) from each of the three stimulus categories was systematically paired with a mild electric stimulation (unconditioned stimulus (US)) during the acquisition phase, whereas the other stimulus (CS−) from each stimulus category was never associated with it. In the following extinction phase, the US was no longer delivered. Skin conductance response (SCR) was measured continuously throughout the entire conditioning procedure. The conditioned response (CR) was operationalised as the differential SCR to the CS+ minus CS− from the same stimulus category^9,16^ and used as an index of learning.

To assess the role of inter-individual differences, we examined the influence of participants’ achievement motivation on Pavlovian aversive learning to goal-relevant vs. goal-irrelevant stimuli. Achievement motivation refers to the need or concern to develop or demonstrate high ability, and to attain a standard of excellence or a success goal.^32,33^ Inter-individual differences in achievement motivation have been reported to affect how individuals appraise the relevance of objects and situations. For instance, when confronted with achievement-related situations, individuals high in achievement motivation have been shown to appraise these situations as more important than did individuals lower in this trait.^34^ Moreover, individuals with a high level of achievement motivation have been reported to be intrinsically motivated to perform a task for its own sake.^35,36^ In light of this evidence and given that the spatial cueing task involves an achievement component related to task performance and success, we inferred that individuals with high achievement motivation would be highly motivated to perform well in this task, thereby attaching higher relevance to the goal-relevant stimuli and lower relevance to the goal-irrelevant stimuli than individuals with lower achievement motivation because of their respective informativeness and instrumentality, or lack thereof, for task accomplishment.

As preferential emotional learning is generally characterised by a faster acquisition of the conditioned response and/or an enhanced resistance to extinction of that conditioned response,^11,12^ these two indicators being considered as equally valid,^37^ we hypothesised that the conditioned response to goal-relevant stimuli would be (a) acquired faster and (b) more resistant to extinction than the conditioned response to goal-irrelevant stimuli. Furthermore, we predicted that inter-individual differences in achievement motivation would modulate the acquisition readiness and the resistance to extinction of the conditioned response to goal-relevant stimuli compared with goal-irrelevant stimuli, with higher achievement motivation leading to a greater difference in the conditioned response to goal-relevant vs. goal-irrelevant stimuli during early acquisition and during extinction.

## Results

### Spatial cueing task

**Fig. 2:**
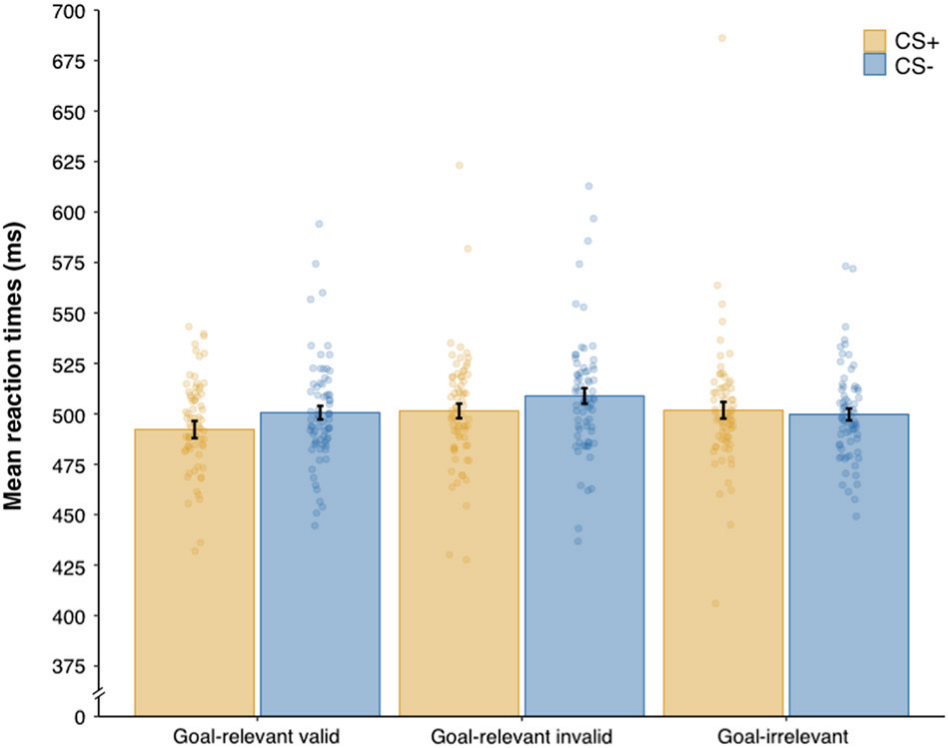
Mean reaction times during the spatial cueing task as a function of stimulus type (to-be-CS+ vs. to-be-CS−) and stimulus category (goal-relevant valid vs. goal-relevant invalid vs. goal-irrelevant). The dots indicate normalised data for individual participants. Error bars indicate ± 1 standard error of the mean adjusted for within-participant designs

The reaction times in the spatial cueing task were analysed using a repeated-measures general linear model (GLM) assuming compound symmetry covariance structure with stimulus type (to-be-CS+ vs. to be-CS−) and stimulus category (goal-relevant valid vs. goal-relevant invalid vs. goal-irrelevant) as within-participant categorical factors, and participants’ standardised (*z*-score) achievement motivation score as a continuous predictor. This analysis revealed a marginal trend for the main effect of stimulus category, *F*(2, 140) = 2.85, *p* *=* 0.061, *η*^2^_p_ = 0.039, 90% confidence interval (CI) [0.000, 0.095]. No other effect was observed (all *F*s < 2.39, all *p*s > 0.12, all *η*^2^_p_s < 0.033). A polynomial contrast analysis showed a statistically significant linear trend in the reaction times as a function of stimulus category, *F*(1, 70) = 5.41, *p* = 0.023, *η*^2^_p_ = 0.072, 90% CI [0.005, 0.181], indicating increased reaction times in detecting the target from goal-relevant valid cues (*M* = 496.52 ms, SD = 129.53) to goal-irrelevant cues (*M* = 500.92 ms, SD = 147.23) to goal-relevant invalid cues (*M* = 505.21 ms, SD = 140.12; Fig. 2). This result reflects the occurrence of a cueing validity effect, hence suggesting that the spatial cueing task triggered attention orienting, although it is important to note that this effect was small.

As descriptive analyses revealed the presence of an outlier in the reaction time data exhibiting slow reaction times in all the conditions, we also performed statistical analyses excluding this outlier. These analyses revealed a statistically significant main effect of stimulus category, *F*(2, 138) = 3.19, *p* = 0.044, *η*^2^_p_ = 0.044, 90% CI [0.0006, 0.103]. No other effect was statistically significant (all *F*s *<* 2.28, all *p*s > 0.10, all *η*^2^_p_s < 0.032). The linear trend remained statistically significant after the outlier exclusion, *F*(1, 69) = 4.33, *p* = .041, *η*^2^_p_ = 0.059, 90% CI [0.001, 0.165].

### Differential Pavlovian aversive conditioning

**Fig. 3:**
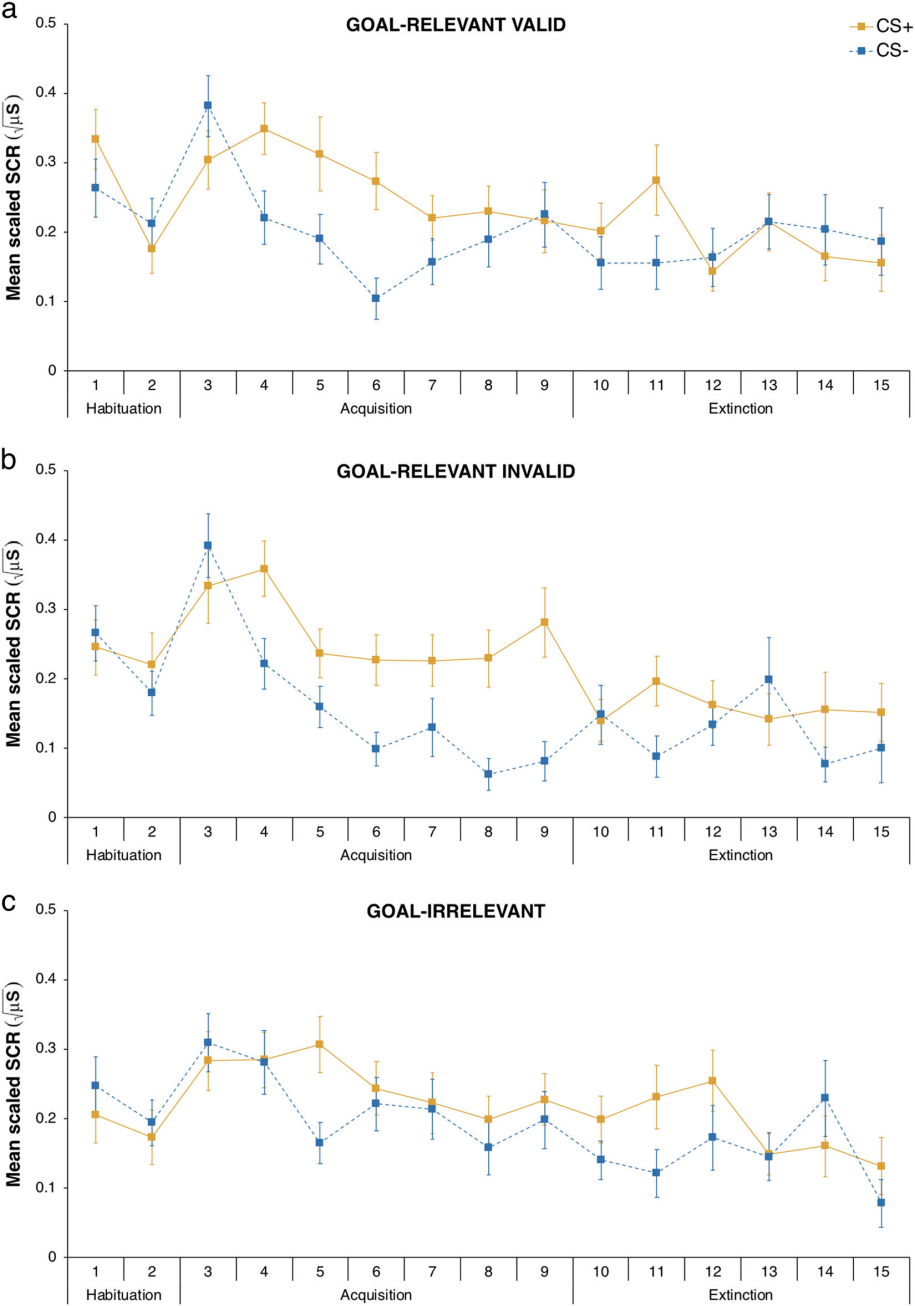
Mean scaled skin conductance response (SCR) to the conditioned stimuli as a function of conditioned stimulus type (CS+ vs. CS−) across trials. Mean scaled SCR to **a** goal-relevant valid stimuli, **b** goal-relevant invalid stimuli, and **c** goal-irrelevant stimuli. Error bars indicate ± 1 standard error of the mean adjusted for within-participant designs

According to standard practice in the human conditioning literature,^9,38^ the SCR data (Fig. 3) was analysed separately for each conditioning phase. The habituation and extinction phases were each analysed with a repeated-measures GLM assuming compound symmetry covariance structure with stimulus category (goal-relevant valid vs. goal-relevant invalid vs. goal-irrelevant) as a within-participant categorical factor and participants’ standardised achievement motivation score as a continuous predictor. To examine the differential CR acquisition readiness as a function of the stimulus’ goal-relevance and the modulatory influence of participants’ achievement motivation thereon, the acquisition phase was split into an early and a late phase, and was analysed using a repeated-measures GLM assuming compound symmetry covariance structure with time (early vs. late) and stimulus category (goal-relevant valid vs. goal-relevant invalid vs. goal-irrelevant) as within-participant categorical factors, and participants’ standardised achievement motivation score as a continuous predictor. During habituation, there were no pre-existing differences in differential SCRs to the various stimulus categories, or as a function of participants’ achievement motivation or the interaction between these factors (all *F*s < 0.48, all *p*s > 0.62, all *η*^2^_p_s < 0.007).

To assess whether a CR was successfully acquired (i.e. greater SCRs to the CS+ than to the CS−) in response to the different stimulus categories (goal-relevant valid vs. goal-relevant invalid vs. goal-irrelevant) during acquisition as expected, we performed one-tailed one-sample *t*-tests on the CR across the entire acquisition phase. These tests showed that the SCRs to the CS+ were larger than to the CS− for goal-relevant valid stimuli, *t*(71) = 3.32, *p* < 0.001 (one-tailed), *g*_av_ = 0.547, 95% CI [0.212, 0.891], and goal-relevant invalid stimuli, *t*(71) = 6.09, *p* < 0.001 (one-tailed), *g*_av_ = 1.005, 95% CI [0.646, 1.380], thereby indicating successful differential conditioning, whereas they were marginally larger than to the CS− for goal-irrelevant stimuli, *t*(71) = 1.49, *p* = 0.070 (one-tailed), *g*_av_ = 0.246, 95% CI [−0.082, 0.577]. The apparent less robust differential conditioning to goal-irrelevant stimuli was mainly driven by the existence of an outlier (−5.66 SD from the mean CR to goal-irrelevant stimuli), who was strongly conditioned to the goal-irrelevant CS-. The one-sample *t*-test excluding this outlier indeed reflected a stronger differential conditioning to goal-irrelevant stimuli, *t*(70) = 2.90, *p* = 0.002 (one-tailed), *g*_av_ = 0.482, 95% CI [0.147, 0.824].

**Fig. 4:**
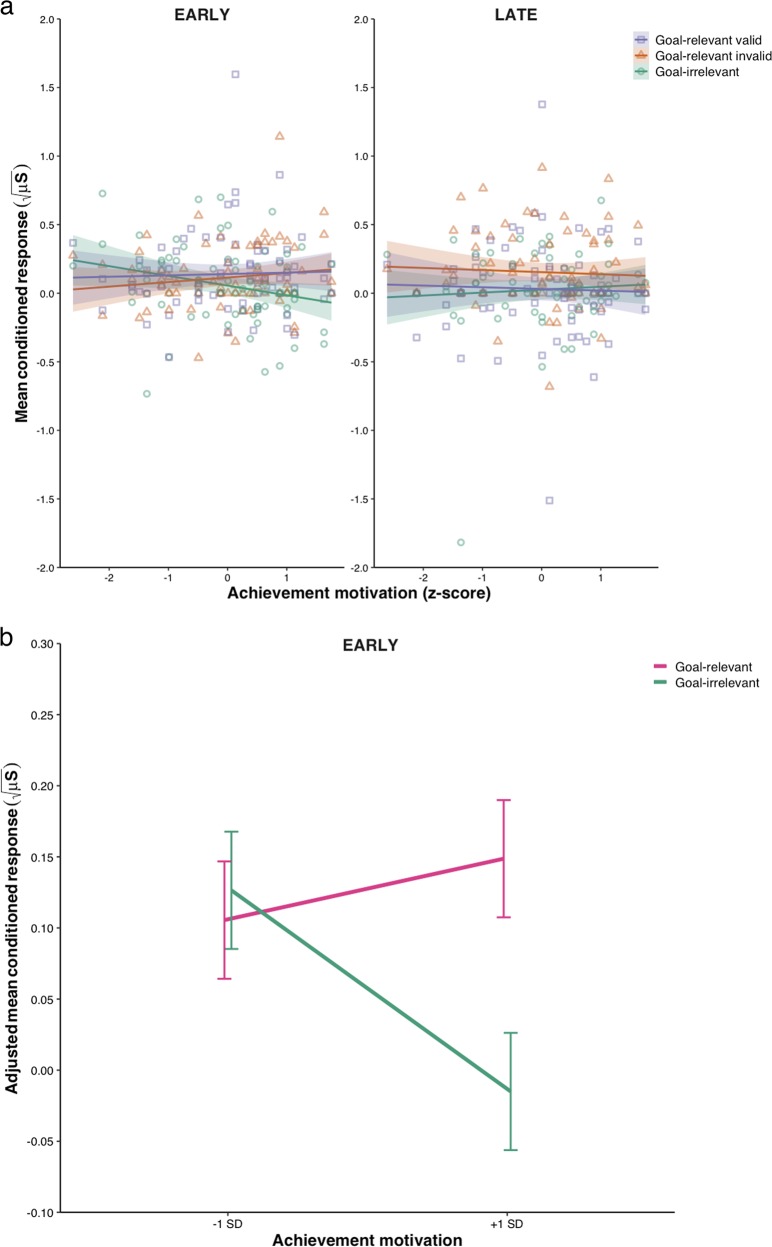
Influence of achievement motivation on the conditioned response to goal-relevant vs. goal-irrelevant stimuli during acquisition. **a** Mean conditioned response as a function of stimulus categories (goal-relevant valid vs. goal-relevant invalid vs. goal-irrelevant) and participants’ standardised (*z*-score) achievement motivation score in the early and the late acquisition phase. The points indicate data for individual participants. The curves represent the best-fitting regression lines using least squares estimation and their 95% confidence interval. **b** Mean adjusted conditioned response to goal-relevant vs. goal-irrelevant stimuli during early acquisition as a function of low (−1 SD) and high (+1 SD) achievement motivation. Error bars indicate ± 1 standard error of the mean

Moreover, the GLM revealed a statistically significant main effect of stimulus category, *F*(2, 140) = 3.81, *p* = 0.024, *η*^2^_p_ = 0.052, 90% CI [0.004, 0.113]. A planned contrast analysis showed that goal-relevant valid (contrast weight: +1) and goal-relevant invalid (contrast weight: +1) stimuli (*M* = 0.11, SD = 0.15) led to the acquisition of a larger CR than goal-irrelevant stimuli (contrast weight: −2; *M* = 0.04, SD = 0.23), *F*(1, 71) = 5.49, *p* = 0.022, *η*^2^_p_ = 0.072, 90% CI [0.006, 0.181]. Albeit not statistically significant, we also observed a marginal trend for the interaction between time and stimulus category, *F*(2, 140) = 2.61, *p* = 0.077, *η*^2^_p_ = 0.036, 90% CI [0.000, 0.090], and for the three-way interaction between time, stimulus category, and achievement motivation, *F*(2, 140) = 2.65, *p* = 0.074, *η*^2^_p_ = 0.036, 90% CI [0.000, 0.091]. No other effect reached statistical significance (all *F*s < 1.14, all *p*s > 0.29, all *η*^2^_p_s < 0.016). To specifically test our a priori hypothesis concerning the CR acquisition readiness to goal-relevant vs. goal-irrelevant stimuli and its modulation by inter-individual differences in achievement motivation, we constructed a contrast comparing the difference between the CR to goal-relevant valid (contrast weight: +1) and goal-relevant invalid (contrast weight: +1) stimuli vs. goal-irrelevant stimuli (contrast weight: −2) during early acquisition, and tested whether this difference was influenced by participants’ standardised achievement motivation score by means of a repeated-measures GLM. Consistent with our prediction, this analysis indicated that the difference between the CR to goal-relevant stimuli and the CR to goal-irrelevant stimuli was modulated by participants’ achievement motivation during early acquisition, *F*(1, 70) = 5.15, *p* = 0.026, *η*^2^_p_ = 0.069, 90% CI [0.004, 0.177], with high level of achievement motivation resulting in a greater difference in CR acquisition readiness between goal-relevant and goal-irrelevant stimuli (Fig. 4a). Further analyses using simple slopes congruently revealed that participants with high achievement motivation (+1 SD) more readily acquired a CR to goal-relevant stimuli (*M* = 0.15) than to goal-irrelevant stimuli (*M* = −0.02), *F*(1, 70) = 8.11, *p* = 0.006, *η*^2^_p_ = 0.104, 90% CI [0.018, 0.222], whereas no statistically significant difference between goal-relevant stimuli (*M* = 0.11) and goal-irrelevant stimuli (*M* = 0.13) was observed for participants with lower achievement motivation (−1 SD), *F*(1, 70) = 0.13, *p* = 0.717, *η*^2^_p_ = 0.002, 90% CI [0.000, 0.048] (Fig. 4b). Achievement motivation conversely did not moderate the difference between the CR to goal-relevant vs. goal-irrelevant stimuli in late acquisition, *F*(1, 70) = 0.62, *p* = 0.433, *η*^2^_p_ = 0.009, 90% CI [0.000, 0.076].

Analysis of the extinction phase showed that the CR did not statistically differ across the three stimulus categories, *F*(2, 140) = 0.54, *p* *=* 0.586, *η*^2^_p_ = 0.008, 90% CI [0.000, 0.037], suggesting a similar CR extinction to goal-relevant valid, goal-relevant invalid, and goal-irrelevant stimuli. The extinction of the CR was likewise not affected by participants’ achievement motivation (all *F*s < 0.32, all *p*s > 0.57, all *η*^2^_p_s < 0.005). The difference between the CR to goal-relevant stimuli and the CR to goal-irrelevant stimuli was not modulated by participants’ achievement motivation either, *F*(1, 70) = 0.15, *p* = 0.696, *η*^2^_p_ = 0.002, 90% CI [0.000, 0.050]. This result reflects that the CR persistence to goal-relevant compared with goal-irrelevant stimuli did not statistically differ as a function of participants’ achievement motivation during extinction.

## Discussion

Altogether, our results show that goal-relevant stimuli induced the acquisition of a larger conditioned response than goal-irrelevant stimuli, thus suggesting stronger Pavlovian aversive conditioning. Most importantly, this effect was notably driven by inter-individual differences in achievement motivation that modulated the acquisition readiness of the conditioned response to goal-relevant stimuli compared with goal-irrelevant stimuli, as revealed by an interaction of the stimulus’ goal-relevance with participants’ achievement motivation during early acquisition. Participants with high achievement motivation more readily acquired a conditioned response to goal-relevant stimuli than to goal-irrelevant stimuli, thus reflecting a learning bias, whereas no learning bias was observed in participants with lower achievement motivation. This indicates that inter-individual differences can produce enhanced Pavlovian aversive conditioning to the very same stimuli depending on their relevance to the individual’s current concerns, such as their achievement motive. Such findings dovetail nicely with the relevance detection model^15,16^ according to which preferential emotional learning stems from the interaction between the stimulus and the organism’s current concerns, thereby assigning a crucial role to inter-individual differences in enhanced emotional learning. On the other hand, we failed to observe an enhanced resistance to extinction to goal-relevant vs. goal-irrelevant stimuli, and no modulatory effect of inter-individual differences in achievement motivation was reported thereon, which is at odds with our predictions.

The fact that we found faster Pavlovian aversive conditioning to goal-relevant vs. goal-irrelevant stimuli in participants high in achievement motivation but not in those lower in achievement motivation may relate to the interplay between the manipulation of stimuli’s relevance for task goals and participants’ current concerns. The construct of goal-relevance has been suggested to cover at least three partly dissociable but related components:^39–41^ (1) task-relevance, which pertains to the degree to which a stimulus signals the opportunity of implementing and satisfying a specific goal in a given task, (2) informativeness, which refers to the degree to which a stimulus provides reliable information about a goal’s satisfaction status, and (3) the impact a stimulus has on the individual’s goals. It has been further advanced that a stimulus that is task-relevant is likewise goal-relevant in terms of informativeness and impact, whereas it can be goal-relevant in terms of informativeness and/or impact without being task-relevant.^40^ Importantly, task-relevance however differs from current concerns in that it refers to task instructions, and begins and ends in task context; in contrast, current concerns involve a state of commitment about their satisfaction that extends across various contexts and situations beyond a particular task.^18,42^ Accordingly, the stimuli’s goal-relevance may have generalised beyond the spatial cueing task for participants with high achievement motivation because of the stimuli’s informativeness and/or impact to their achievement-related concerns, whereas it ended with the spatial cueing task for participants with lower achievement motivation, the goal-relevant stimuli no longer being task-relevant and of higher relevance to their current concerns than the goal-irrelevant stimuli.

Critically, the facilitated Pavlovian aversive conditioning to goal-relevant than to goal-irrelevant stimuli observed in participants with high achievement motivation furthermore suggests that stimuli that are detected as relevant to the organism’s concerns can also be readily conditioned to threat even though they hold no intrinsic biological evolutionary significance. This finding reflects that preferential emotional learning is not restricted to stimuli that are relevant in a phylogenetic sense. In this respect, the current study concurs with previous research on human conditioning reporting enhanced Pavlovian aversive learning to ontogenetic threat-relevant stimuli.^19,20^ It even adds to these earlier reports by showing that initially neutral stimuli devoid of any pre-existing threat value that have acquired goal-relevance can likewise be readily associated with a naturally aversive event in individuals high in achievement motivation. In that sense, our results suggest that preferential emotional learning may be underlain by a relevance detection mechanism, as opposed to a fear- or threat-specific mechanism, allowing the organism to adaptively and flexibly produce a learning bias towards specific stimuli depending on their relevance to the organism’s current concerns.^15,16^

Nonetheless, the fact that we did not find effects of stimulus’ goal-relevance during extinction suggests that the preferential aversive learning to goal-relevant stimuli as a function of inter-individual differences in achievement motivation was rather modest and transient. This negative finding notably departs from the greater resistance to extinction to threat-relevant stimuli than to threat-irrelevant stimuli typically reported in the human conditioning literature.^10,11^ Although the present experiment indicates that goal-relevant stimuli can produce facilitated Pavlovian aversive conditioning relative to goal-irrelevant stimuli even if they have no inherent threat value when considering achievement motivation, it appears that the effects of goal-relevance observed therein are likely to be smaller than those usually obtained with threat-relevant stimuli. It is worth noting, however, that such potential difference is fully consistent with our general framework supporting the relevance detection hypothesis: whereas threat-relevant stimuli are highly relevant for the organism’s survival, the goal-relevant stimuli used here were only temporarily relevant for task-related goals in laboratory settings. In other words, because survival is arguably one of the highest prioritised concerns, survival-relevance can be conceptually considered as a high-value sub-category of goal-relevance. Accordingly, the type of goal-relevant stimuli that we used in the current study probably held a lower level of relevance to the organism than threat-relevant stimuli, thereby possibly accounting for the occurrence of seemingly weaker effects. In this context, an interesting avenue for future research would thus be to directly compare the impact of survival-relevance (e.g. using threat-relevant stimuli) to the impact of other types of goal-relevance on Pavlovian aversive conditioning, while ideally using goal-relevant stimuli of comparable relevance to that of threat-relevant stimuli.^16^ Our framework would also predict that individual differences in specific survival-relevant concerns would cause various degrees of preferential aversive learning to threat-related stimuli.

Relatedly, the lack of differential resistance-to-extinction effects may tentatively be imputed to the specifics of our manipulation of goal-relevance. In particular, the use of a spatial cueing task in which the cues were presented exogenously for a brief amount of time (100 ms) may have precluded participants from forming an explicit and strong knowledge of the associations between the cues and the stimulus categories, and mainly tapped into implicit processes. Consistent with this proposition, the subjective ratings (see Supplementary Methods) suggested that participants did not discriminate the differential predictive value of the different stimuli used as cues during the spatial cueing task. In this context, the relevance manipulation was probably too weak to induce long-lasting effects that could as well influence the persistence of the conditioned response. Future studies are therefore needed to assess whether a stronger relevance manipulation, for instance by using an endogenous cueing task allowing participants to integrate information about the stimuli’s goal-relevance at a more explicit, controlled level,^43^ could lead to a differential resistance-to-extinction effect for goal-relevant stimuli compared with goal-irrelevant stimuli, besides faster Pavlovian aversive learning.

Whereas our manipulation of goal-relevance by means of a spatial cueing task was probably subtle, the subjective ratings collected after extinction (see Supplementary Methods) clearly reflected that participants were aware of the contingencies between the conditioned stimuli and the unconditioned stimulus, and that the conditioning procedure elicited robust evaluative effects, the CSs+ being evaluated as less pleasant, more arousing, and more relevant than the CSs− (see Supplementary Figure 1). Presumably, this potent conditioning procedure may have overshadowed “residual” relevance effects produced by the preceding spatial cueing task, the salience of the CSs+ association with an electric stimulation prevailing over the stimuli’s previously acquired goal-relevance, especially during the extinction phase. This too could potentially account for the fact that we observed faster Pavlovian aversive conditioning to goal-relevant stimuli than to goal-irrelevant stimuli in participants high in achievement motivation, but did not find differential extinction effects as a function of stimulus’ goal-relevance and achievement motivation.

As goal-relevant stimuli have been shown to attract attention,^18,44^ it is possible that the goal-relevant stimuli induced facilitated acquisition of a conditioned response in participants high in achievement motivation because more attention was allocated to them than to the goal-irrelevant stimuli. Given that the goal-relevant stimuli were also highly predictive with respect to target location in the spatial cueing task while the goal-irrelevant stimuli were associated with a high uncertainty, this suggestion aligns with the Mackintosh’s^45^ attentional model of Pavlovian conditioning. According to this model, the amount of attention devoted to the conditioned stimulus is a core determinant of learning, with predictive stimuli being better attended and hence more readily conditioned. In this light, attention could provide an underlying mechanism contributing to the occurrence of learning bias to goal-relevant stimuli in participants high in achievement motivation, thus possibly mirroring the contribution of attention to the enhancement effects of emotion on memory for instance.^46^

Further consideration of the role of predictiveness and uncertainty additionally raises the question of whether these constructs may have influenced our findings. Predictiveness and uncertainty have been shown to affect associative learning and attentional processes,^45,47–49^ in particular through their impact on stimulus’ salience^45,49,50^ or informativeness,^41^ as well as are considered as an important evaluation criterion for determining the relevance of a stimulus in appraisal theories.^14^ Although the cues’ predictiveness and/or uncertainty may have had a general influence on their appraised relevance and contributed to our findings, it seems unlikely that our results were solely driven by these factors. Indeed, it remains unclear to what extent such an account can accommodate the observed effects of inter-individual differences in achievement motivation on the acquisition readiness of the conditioned response to goal-relevant vs. goal-irrelevant stimuli, without requiring the involvement of additional explanatory mechanisms directly tied to the organism’s achievement-related concerns. Accordingly, it appears that goal-relevance offers a more parsimonious and plausible key mechanistic explanation of our findings. Further research would nevertheless be necessary to disentangle the specific contributions of predictiveness and/or uncertainty and of goal-relevance to faster Pavlovian aversive conditioning, for instance by implementing a paradigm enabling the orthogonalisation of these factors.^41^

Considering that our sample mostly consisted of women participants, we cannot be sure that our results can generalise to men, which represents a limitation of our study. As women and men can differ in conditioned threat acquisition,^51^ it could be possible that the modulation of Pavlovian aversive conditioning to goal-relevant vs. goal-irrelevant stimuli may have been affected by sex differences in achievement motivation. However, women (*M* *=* 4.04, SD = 0.82) and men (*M* = 4.32, SD = 0.75) participants in our sample did not statistically differ in achievement motivation scores, as reflected by a Welch’s *t*-test for unequal sample sizes, *t*(18.79) = −1.16, *p* = 0.262, *g*_s_ = −0.332, 95% CI [−0.956, 0.250]. This result thereby provides no evidence that sex differences in achievement motivation influenced our results. Another caveat relates to the fact that we did not consider the role of the hormonal cycle stage of our women participants, which has been shown to affect skin conductance response, notably during extinction learning.^51^ Although we cannot exclude the possibility that this factor may have had an effect on our results, we are not aware of any empirical evidence suggesting that the hormonal cycle stage specifically facilitates the acquisition of a conditioned response to certain categories of stimuli, such as goal-relevant stimuli in the present case, relative to other stimulus categories, such as goal-irrelevant stimuli.

In sum, our study suggests that stimuli without any inherent biological evolutionary significance but temporarily associated with a higher goal-relevance can also induce facilitated Pavlovian aversive learning provided that specific individual motivation dispositions are met concurrently, thus reflecting that the occurrence of a learning bias is crucially dependent on inter-individual differences in the organism’s current concerns. In the present case, the learning bias towards goal-relevant stimuli in comparison with goal-irrelevant stimuli was expressed as a greater conditioned response acquisition, and, importantly, as a facilitated conditioned response acquisition in participants scoring high on achievement motivation, whereas no effect on the persistence of the conditioned response was observed. Although the impact of goal-relevance was modest and transient, these findings lean towards the view that Pavlovian aversive conditioning may be driven by a general mechanism of relevance detection that is not necessarily selective for stimuli holding a pre-existing threat value.^16^ This mechanism yields flexibility in the way specific stimuli encountered in the environment are eventually learned preferentially, depending primarily on the complex interplay between the stimulus at hand and the organism’s current concerns. Hence, relevance detection provides a flexible theoretical framework that can not only incorporate the extant evidence on preferential Pavlovian aversive learning in the human conditioning literature but also account for the large inter-individual differences typically observed in human emotional learning. In this perspective, the relevance detection approach holds promise for contributing to an improved mechanistic understanding of emotional learning in humans. Ultimately, this alternative framework could also contribute to unravelling emotional learning impairments preceding or following the onset and maintenance of specific affective disorders, such as anxiety disorders and phobias, thus hopefully aiding in developing and validating new individualised and targeted interventions for these conditions.

## Methods

### Participants

Eighty-eight participants took part in the experiment, which was approved by the Faculty of Psychology and Educational Sciences ethics committee at the University of Geneva. All ethical regulations were complied with. Sixteen participants were excluded from the analyses based on predetermined criteria:^9,15,16^ seven because of technical problems, three for displaying virtually no SCR, and six for failing to acquire a conditioned response to at least one of the conditioned stimuli predictive of the unconditioned stimulus. The final sample size consisted of 72 participants (59 women), aged between 18 and 70 years old (mean age = 22.67 ± 7.58 years).

We established the sample size prior to data collection by means of a power analysis conducted with G*Power 3 (ref. ^52^), which indicated that a total sample of 71 participants would be required to obtain a power of 80% to detect a relatively small effect (*d* = 0.3) as reported in a previous study.^19^ For counterbalancing purposes, we sought to recruit a sample of 72 participants that were conditioned to at least one of the three stimulus categories, and stopped data collection when the required number of participants had been reached.

### Stimuli and apparatus

Six neutral complex geometric figures commonly used in human conditioning paradigms^31,53^ served as cues in the spatial cueing task and subsequently as conditioned stimuli (CS) in the Pavlovian differential aversive conditioning paradigm. The geometric figures were divided into three stimulus categories as a function of their goal-relevance and predictive power of target location in the spatial cueing task: (a) the goal-relevant valid stimuli, which consistently predicted target location, (b) the goal-relevant invalid stimuli, which consistently predicted the opposite location relative to the target, and (c) the goal-irrelevant stimuli, which were nonpredictive of target location by predicting target location and the opposite location with an equal probability (50%). The goal-relevant valid and the goal-relevant invalid geometric figures allowed participants to anticipate target location, and were therefore relevant for the spatial cueing task goals. By contrast, the goal-irrelevant geometric figures were uninformative about upcoming target location, thus being irrelevant for the spatial cueing task. We used two types of goal-relevant stimuli in order to be able to dissociate a general effect of goal-relevance from a mere cue (in)validity effect. The attribution of the stimulus categories to the six geometric figures were counterbalanced across participants. In the differential Pavlovian aversive conditioning procedure, one geometric figure from each of the three stimulus categories served as a CS+, whereas the other one served as a CS−; this assignment being counterbalanced across participants. The unconditioned stimulus (US) was a mild electric stimulation (200-ms duration, 50 pulses/s) delivered to the participants’ nondominant wrist through a Grass SD9 stimulator (Grass Medical Instruments, West Warwick, RI) charged by a stabilised current.

The conditioned response (CR) was assessed through SCR measured with two Ag-AgCl electrodes (6-mm contact diameter) filled with 0.5% NaCl electrolyte gel. The electrodes were attached to the distal phalanges of the index and middle fingers of the participants’ nondominant hand. The SCR data was continuously recorded at 1000 Hz with a BIOPAC MP150 system (Santa Barbara, CA) and analysed offline with AcqKnowledge software (Version 4.2; BIOPAC Systems Inc., Goleta, CA).

### Procedure

Upon arrival at the laboratory, participants were informed about the general procedure of the experiment and provided written informed consent. They next performed the spatial cueing task. Participants were then asked to rate the geometric figures on several dimensions (see Supplementary Methods) before undertaking the differential Pavlovian aversive conditioning procedure. After the end of the conditioning procedure, they were again asked to provide subjective ratings of the geometric figures (see Supplementary Methods). Finally, participants completed the Unified Motive Scales^54^ (UMS) to measure their achievement motivation.

### Spatial cueing task

In this task^31^ (Fig. 1), each trial started with a fixation cross presented for a duration randomly varying between 250 and 750 ms. A cue was subsequently presented either on the left or the right side of the fixation cross for 100 ms. The cues consisted of the six geometric figures, divided into the three stimulus categories (i.e. two goal-relevant valid cues, two goal-relevant invalid cues, and two goal-irrelevant cues). Following a brief interval after the cue was removed (blank screen; 16.7 ms), a target consisting of a black bar was presented onscreen for 100 ms. Participants were requested to press as quickly and accurately as possible with the second digit of their dominant hand the “B” key when the target was displayed horizontally and the “N” key when it was displayed vertically, and their reaction times and accuracy were measured. The target appeared either at the same location as the cue (valid trial) or at the opposite location (invalid trial; Fig. 1). After participants’ response, each trial ended with an intertrial interval randomly varying between 700 and 1300 ms. Participants were asked to look at the fixation cross during the entire task.

Participants first undertook a training session of 24 trials. Each of the six cues was presented four times. The training session was repeated until participants reached an accuracy of 75%, after which the experimental task started. It was composed of 144 trials, divided into 48 trials for each stimulus category, each cue being presented 24 times. During both the training session and the experimental task, the valid and invalid trials were equally presented, and the left or right position of the cue and the target, as well as the horizontal and vertical orientation of the target, were counterbalanced, and the order of the trials pseudorandomised. All responses that were incorrect (4.06% of the trials), faster than 200 ms (0.09% of the trials), or more than three standard deviations from the participant’s mean (1.63% of the trials) were removed prior to analysis.^31^

### Differential Pavlovian aversive conditioning

Before conditioning, the electrodes for measuring SCR were placed on participants and a work-up procedure was conducted to individually set the electric stimulation intensity (*M* *=* 33.73 V, SD = 9.48, range = 10–50 V) to a level reported as “uncomfortable, but not painful”. During the initial habituation phase, each of the six geometric figures serving as CSs was presented twice without being reinforced. In the following acquisition phase, each CS was presented seven times. This phase always began with a CS+ trial. Five of the seven presentations of each CS+ coterminated with an electric stimulation delivery, whereas the CSs- were never paired with the US. In the extinction phase, each CS was presented six times and the US was no longer delivered. During all the conditioning phases, the CSs were presented for 6 s with a variable intertrial interval ranging from 12 to 15 s. The CSs’ order of presentation was pseudorandomised into 12 different orders.

### Unified motive scales (UMS)

At the end of the experiment, participants filled out the UMS.^54^ This questionnaire offers an explicit measure of individuals’ motives. It is composed of 54 items measured on a 6-point Likert scale ranging from 1 (strongly disagree) to 6 (strongly agree). These items assess various types of motivation, including achievement motive, power motive, affiliation motive, intimacy motive, fear of losing control, fear of failure, fear of rejection, and fear of losing emotional contact.^54^ Given our a priori hypotheses, we exclusively focused on the achievement motive subscale, which comprised 10 items (standardised Cronbach’s *α* = 0.85). Each participant’s responses to these items were averaged to compute their achievement motivation score (*M* = 4.09, SD = 0.81, range = 2.0–5.5; see Supplementary Figure 2).

### Response definition

SCR was scored for each trial as the peak-to-peak amplitude difference in skin conductance of the largest response starting in the 0.5–4.5 s temporal window following CS onset. The minimal response criterion was 0.02 µS. Responses below this criterion were scored as zero and remained in the analysis. Before analysis, the SCR data was low-pass filtered (Blackman −92 dB, 1 Hz). SCRs were detected automatically with AcqKnowledge software and manually checked for artefacts and response (mis)detection. Trials containing artefacts influencing the coding of event-related SCRs (<0.001%) were omitted from the analyses. The raw SCRs were square-root-transformed to normalise the distributions and scaled according to each participant’s mean square-root-transformed unconditioned response (UR). The UR was scored as the peak-to-peak amplitude difference in skin conductance of the largest response starting in the 0.5–4.5 s temporal window after the US delivery. The habituation means were composed of the first two presentations of each CS. To investigate the CR acquisition readiness, the acquisition means were split into an early (i.e. the first three presentations of each CS subsequent to the first pairing between the CS+ from the stimulus category and the US) and a late (i.e. the following three presentations of each CS) phase.^15,16,38^ Because the CSs+ became predictive of the US solely after their first association with the electric stimulation, the first acquisition trial for each CS was removed from the analyses. The extinction means included the last six presentations of each CS. The conditioning data analyses were performed on the CR, which was computed as the SCR to the CS+ minus the SCR to the CS− from the same stimulus category.^9,15,16^

### Statistical analyses

Statistical analyses were performed with R^55^ and the afex package.^56^ An alpha level of 0.05 was adopted for all the analyses performed. When descriptive analyses revealed the presence of outliers (value smaller than the lower quartile minus three times the interquartile range, or value larger than the upper quartile plus three times the interquartile range^57^), we conducted the analyses including and excluding the outliers, and report the outcome of both analyses when the outlier removal altered statistical significance. Otherwise, we only report the results of the analyses including the outliers. We report *η*^2^_p_ or Hedges’ *g*_av_ and their 90 or 95% confidence interval, respectively, as estimates of effect sizes.

### Reporting Summary

Further information on research design is available in the Nature Research Reporting Summary linked to this article.

## Supplementary information

Supplementary Information

Life Sci Reporting Summary

## Data Availability

The datasets generated and analysed during the current study, as well as the materials used therein, are available in the Open Science Framework repository at 10.17605/OSF.IO/EQA6S.

## Supplementary information

**Supplementary information** accompanies the paper on the *npj Science of Learning* website (10.1038/s41539-019-0043-3).

## Code availability

The code used for data analysis can be found in the Open Science Framework repository at 10.17605/OSF.IO/EQA6S.
