# Supplementary material for: Achievement motivation modulates Pavlovian aversive conditioning to goal-relevant stimuli
Source: NPJ Sci Learn. 2019 Apr 24;4:4. doi: 10.1038/s41539-019-0043-3 (PMC6482202; doi:10.1038/s41539-019-0043-3)
Supplement: Supplementary file 1 — Supplementary Information [file 41539_2019_43_MOESM1_ESM.pdf]

Supplementary Information

Achievement motivation modulates Pavlovian aversive conditioning to goal-relevant stimuli

by Y. Stussi, A. Ferrero, G. Pourtois, and D. Sander

Supplementary Methods

Subjective ratings

Subsequent to the spatial cueing task, participants completed subjective ratings of the geometric figures' (a) predictive power, (b) liking, (c) arousal, and (d) relevance. In this procedure, the geometric figures were presented to participants, accompanied by a visual analogue scale (VAS). For the prediction power ratings, participants were asked to rate to what extent the geometric figure was predictive of target location on the same side from 0 (*never*) to 100 (*always*). For the liking ratings, they were asked to rate to what extent the geometric figure was unpleasant or pleasant from 0 (*very unpleasant*) to 100 (*very pleasant*). For the arousal and the relevance ratings, participants were asked to rate to what extent the geometric figure was arousing from 0 (*not at all arousing*) to 100 (*very arousing*), and to what extent it was important to them from 0 (*not at all important*) to 100 (*very important*), respectively.

After the extinction phase of the conditioning procedure, participants completed (a) CS-US contingency ratings, along with (b) liking, (c) arousal, and (d) relevance ratings of the geometric figures. For the CS-US contingency ratings, they were asked to rate to what extent the geometric figure was predictive of the delivery of an electric stimulation on a VAS going from 0 (*never*) to 100 (*always*). The procedure for the liking, arousal, and relevance ratings was identical to the one used in the preconditioning ratings. The order of the geometric figure presentations and the questions was randomised across participants for both the

preconditioning and the postconditioning subjective ratings. Finally, participants rated to what extent it was important to them to perform well in the spatial cueing task on a VAS ranging from 0 (*not at all important*) to 100 (*very important*; $M = 74.87$, $SD = 15.88$, range = 17.05-100).

The preconditioning ratings of the stimuli's predictive power were analysed with a repeated-measures general linear model (GLM) assuming compound symmetry covariance structure including stimulus type (CS+ vs. CS-) and stimulus category (goal-relevant valid vs. goal-relevant invalid vs. goal-irrelevant) as within-participant categorical factors, and participants' standardised (z-score) achievement motivation score as a continuous predictor. This analysis showed that there was no statistical difference across the three stimulus categories, or as a function of stimulus type, achievement motivation, or the interaction between any of these factors in the predictive power ratings of the stimuli (all Fs < 1.07, all ps > .34, all η^2_ps < .016; Supplementary Figure 1a). These results tentatively suggest that participants did not seem to be able to explicitly distinguish the predictive power of the different stimuli used as cues during the spatial cueing task.

The liking, the arousal, and the relevance ratings were each analysed using a repeated-measures GLM assuming compound symmetry covariance structure including stimulus type (CS+ vs. CS-), stimulus category (goal-relevant valid vs. goal-relevant invalid vs. goal-irrelevant), and time (pre vs. post) as within-participant categorical factors, and participants' standardised achievement motivation score as a continuous predictor. Analysis of the liking ratings revealed statistically significant main effects of stimulus type, $F(1, 70) = 10.18$, $p = .002$, $\eta^2_p = .127$, 90% CI [.029, .249], and of time, $F(1, 70) = 12.27$, $p < .001$, $\eta^2_p = .149$, 90% CI [.042, .274]. These main effects were however qualified by the interaction between stimulus type and time, $F(1, 70) = 20.09$, $p < .001$, $\eta^2_p = .223$, 90% CI [.093, .350]. Follow-up simple effects analyses indicated that there was no statistical difference in the

preconditioning liking ratings of the CSs+ and the CSs-, $F(1, 70) = 0.15$, $p = .696$, $\eta^2_p = .002$, 90% CI [.000, .050], whereas the CSs+ were rated as less pleasant than the CSs- after conditioning, $F(1, 70) = 17.00$, $p < .001$, $\eta^2_p = .195$, 90% CI [.072, .322] (Supplementary Figure 1b). No other effect reached statistical significance (all Fs < 3.13, all ps > .08, all η^2_ps < .043).

For the arousal ratings, the analysis showed a statistically significant main effect of stimulus type, $F(1, 70) = 53.43$, $p < .001$, $\eta^2_p = .433$, 90% CI [.285, .541], which was qualified by the higher-order interaction between stimulus type and time, $F(1, 70) = 65.29$, $p < .001$, $\eta^2_p = .483$, 90% CI [.338, .584]. Simple effects analysis showed that participants did not statistically differ in their subjective ratings of the CSs+ and the CSs- arousal value before conditioning, $F(1, 70) = 0.95$, $p = .334$, $\eta^2_p = .013$, 90% CI [.000, .087], but deemed the CSs+ more arousing than the CSs- after it, $F(1, 70) = 75.92$, $p < .001$, $\eta^2_p = .520$, 90% CI [.380, .615] (Supplementary Figure 1c). No other effect was found for the arousal ratings (all Fs < 3.15, all ps > .08, all η^2_ps < .044).

The relevance ratings analysis revealed main effects of stimulus type, $F(1, 70) = 15.87$, $p < .001$, $\eta^2_p = .185$, 90% CI [.065, .311], and of time, $F(1, 70) = 14.36$, $p < .001$, $\eta^2_p = .170$, 90% CI [.055, .296]. These main effects were however qualified by their interaction, $F(1, 70) = 18.42$, $p < .001$, $\eta^2_p = .208$, 90% CI [.082, .335]. Further simple effects analyses showed no statistical difference in relevance ratings between the CSs+ and the CSs- prior to conditioning, $F(1, 70) = 0.96$, $p = .330$, $\eta^2_p = .014$, 90% CI [.000, .087], whereas the CSs+ were evaluated as more relevant than the CSs- after it, $F(1, 70) = 19.67$, $p < .001$, $\eta^2_p = .219$, 90% CI [.090, .346] (Supplementary Figure 1d). A statistically significant three-way interaction between stimulus category, time, and participants' achievement motivation was additionally found, $F(2, 140) = 3.41$, $p = .036$, $\eta^2_p = .046$, 90% CI [.002, .106]. Follow-up simple slopes analyses revealed a marginal trend for the simple interaction effect of stimulus

category and time in participants high in achievement motivation (+1 *SD*), $F(2, 140) = 2.64$, $p = .075$, $\eta^2_p = .036$, 90% CI [.000, .091], reflecting that these participants evaluated the goal-relevant invalid ($p = .028$) and the goal-irrelevant ($p < .001$), but not the goal-relevant valid ($p = .186$), stimuli as more relevant after than before conditioning, whereas no simple interaction effect was observed in participants lower in achievement motivation (-1 *SD*), $F(2, 140) = 1.18$, $p = .311$, $\eta^2_p = .017$, 90% CI [.000, .057]. No other effect yielded statistical significance (all *F*s < 3.28, all *p*s > .06, all η^2_ps < .045).

A repeated-measures GLM assuming compound symmetry covariance structure including stimulus type (CS+ vs. CS-) and stimulus category (goal-relevant valid vs. goal-relevant invalid vs. goal-irrelevant) as within-participant categorical factors, and participants' standardised achievement motivation score as a continuous predictor was used to analyse the postconditioning CS-US contingency ratings. This analysis showed that the CSs+ were deemed more likely to be predictive of the US than the CSs-, $F(1, 70) = 164.38$, $p < .001$, $\eta^2_p = .701$, 90% CI [.599, .763] (Supplementary Figure 1e). They conversely did not statistically differ as a function of stimulus category or participants' achievement motivation, and no interaction effect was observed (all *F*s < 2.21, all *p*s > .11, all η^2_ps < .031).

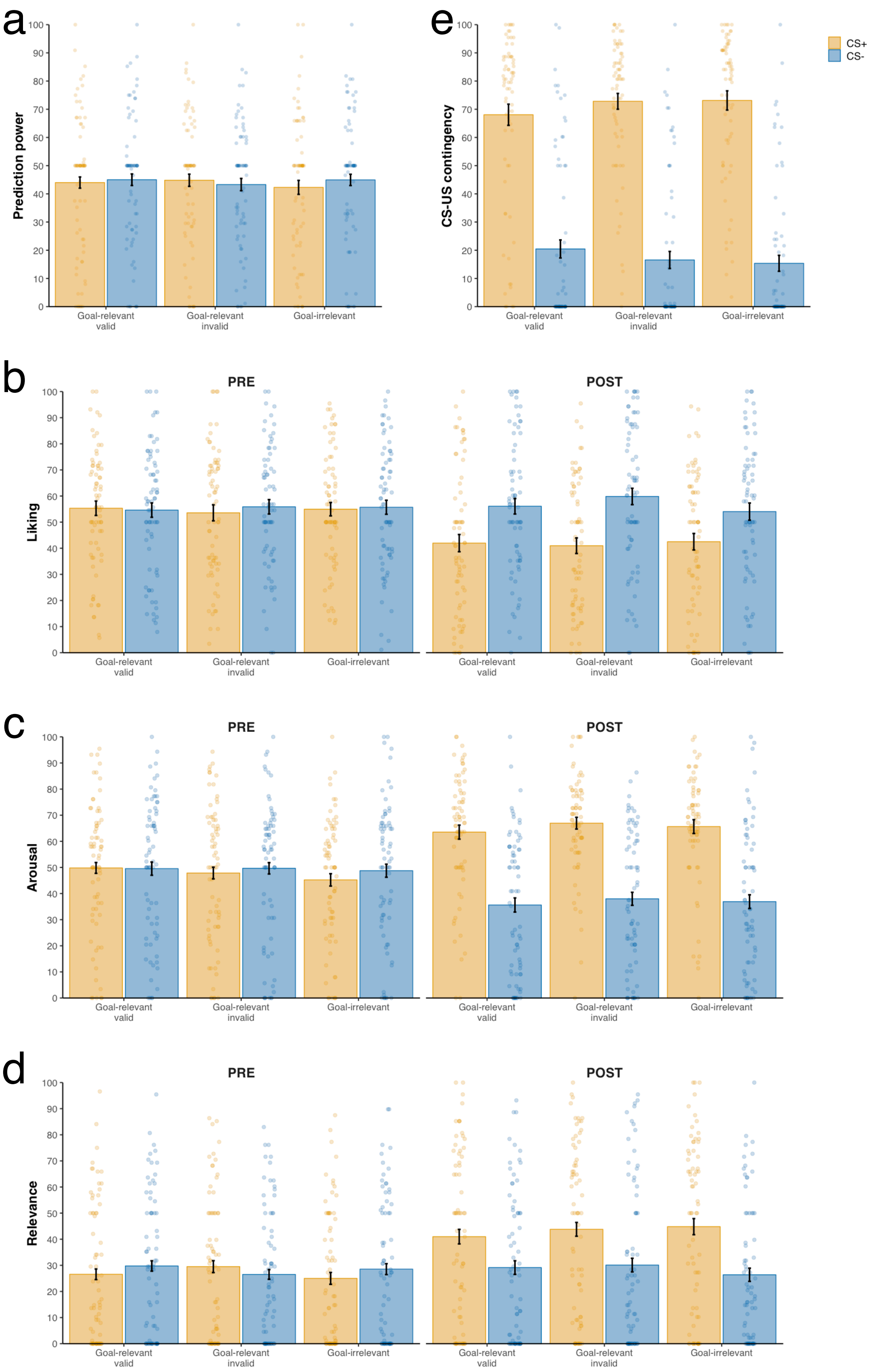
a
Prediction power
Goal-relevant valid
Goal-relevant invalid
Goal-irrelevant
e
CS-US contingency
CS+
CS-
b
PRE
POST
Liking
c
Arousal
d
Relevance

Supplementary Figure 1. Mean subjective ratings before (pre) and after (post) the conditioning procedure as a function of conditioned stimulus type (CS+ vs. CS-) and stimulus category (goal-relevant valid vs. goal-relevant invalid vs. goal-irrelevant). Mean (a) prediction power ratings, (b) liking ratings, (c) arousal ratings, (d) relevance ratings, and (e) CS-US contingency ratings. The dots indicate data for individual participants. Error bars indicate ± 1 standard error of the mean adjusted for within-participant designs.

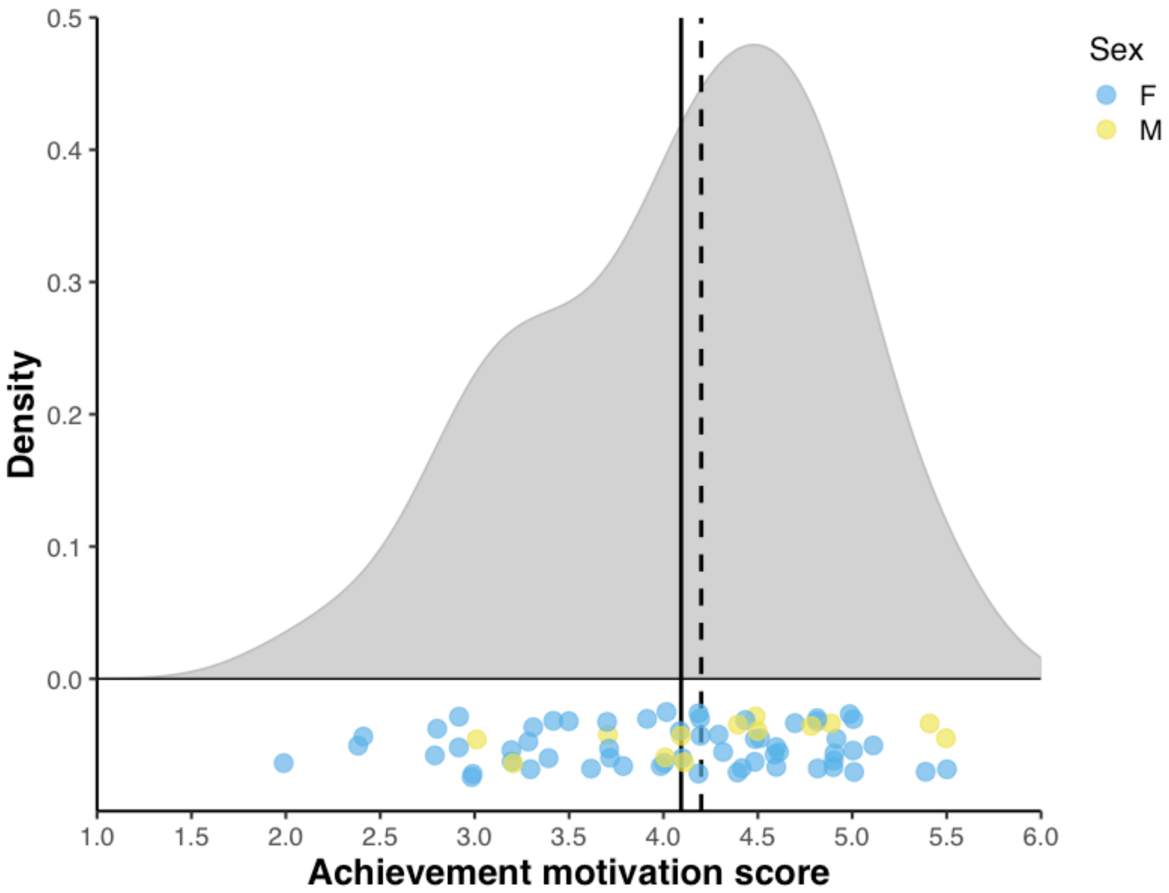

Supplementary Figure 2. Distribution of achievement motivation scores as measured with the Unified Motive Scales (Supplementary ref. 1). The dots indicate data for individual participants. The solid line indicates the mean achievement motivation score, and the dashed line indicates the median achievement motivation score.

Supplementary References

1. Schönbrodt, F. D. & Gerstenberg, F. X. R. An IRT analysis of motive questionnaires: The Unified Motive Scales. *J. Res. Pers.* **46**, 725-742 (2012).
